# Trends in Neuropediatric Physical Therapy

**DOI:** 10.3389/fpubh.2013.00005

**Published:** 2013-04-05

**Authors:** Eduardo B. Neves

**Affiliations:** ^1^Department of Physical Therapy, Campos de Andrade University CenterCuritiba, Paraná, Brazil; ^2^Biomedical Engineering Master Program, Federal Technological University of ParanáCuritiba, Paraná, Brazil

## Introduction

Among the various diseases that cause neuromotor deficits in children, the chronic non-progressive encephalopathy [cerebral palsy (CP)] is one of the most frequent. Children affected by PC may present: sensory disorders, cognitive disorders, abnormal patterns of posture, and postural tone change (1). The literature has signaled some protocols which seem to enhance the neuromotor rehabilitation of these children.

## Physical Therapy Protocols

The aims of the Physical Therapy are to promote and restore body’s functionality. The child with neuromotor deficits uses compensatory mechanisms to overcome the power of gravity. Thus, the repetition in the realization of such compensation creates muscle imbalances, deformities, increased hypertonia, and impair the child’s functionality (2). Therefore, during the rehabilitation process is very important that the therapist keeps the children in a correct posture to facilitate the correction of their body image.

In this sense, some intensive physiotherapy protocols associated with the use of special clothes (dynamic splint) have been shown to treat these children, among them are: the PediaSuit, the TheraSuit, the PenguinSuit, and AdeliSuit (3–5). The special clothes usually consist of: vest, shorts, kneepads, and shoes fitted with hooks and elastic cords that help position the body in an appropriate physical alignment to perform daily activities (5). The recommendation of special clothes is justified because usually the child with CP presents axial hypotonia (mainly trunk) and spasticity in the muscles of appendicular skeleton (limbs) (2).

Apart from the special clothes, the treatment protocols mentioned above are developed in intensive modules (3–4 h per day, 5 days a week during 4–5 weeks). The combination of these features has achieved significant results in rehabilitation process of children with neuromotor deficits.

Among the therapeutic resources used in these protocols can be cited: warm up (including massage, stretching, exercise with passive, active, and active-assisted mobilization); kinesiotherapy (kinesiotherapy cage with active resistance); kinesiotherapy using special clothes (with elastic cage, plank balance, ball, roller, trampoline); respiratory physiotherapy, gait training (using clothes on uneven terrain, stairs, ramps, conveyor, grass, parallel bars with and without obstacles), fine motor activities (use of clay, drawing with pencil, paint brush, glue, engaging games, manipulating objects, and toys), cranio-sacral osteopathy, hydrotherapy, and taping (3–5). Some resources such as: elliptical ergometer adapted for kids and electrical stimulation systems with closed loop and biofeedback could also be part of these protocols, but are still rare in clinical practice.

Bailes et al. (6) conducted a study, in 2011, in which examined the effects of suit wear during an intensive therapy program on motor function among children with CP. Twenty children were randomized in two groups, an experimental (TheraSuit) and a control (control suit) group, and participated in an intensive therapy program. The Gross Motor Function Measure (GMFM) and Pediatric Evaluation of Disability Inventory (PEDI) were administered before and after (4 and 9 weeks). This was the first study to examine the different components of suit therapy. Although the study did not find statistical differences (*p* < 0.05) between the two suits used, in the evaluation by the GMFM and the PEDI, the experimental group (TheraSuit) showed statistically significant improvement after 9 weeks of therapy. This study has several limitations related to the small sample size and lack of blinding of families and treating therapists.

These intensive physiotherapy protocols with use of suits were developed a few years and, therefore, there are few studies on their effectiveness. As for scaling up treatment, this is limited to training therapists in the protocol and the price charged to families, ranging from $ 2000 to $ 4000 for 4 weeks of therapy. However, it has been observed in clinical practice a high demand for these protocols, result of the satisfaction of the families that have already verified its effectiveness.

## Functional Diagnosis and Monitoring of Results

Classics assessment instruments can be suggested for functional diagnosis and monitoring of results achieved in the rehabilitation process of children with neuromotor deficits: the Alberta Infant Motor Scale (AIMS) that identify developmental delays in children under 18 months of age; the PEDI that evaluates functional performance of children between 6 months and 7 years; the GMFM that quantifies the motor function percentage of child in relation to typical motor development of a 5-years-old child; and the Gross Motor Function Classification System (GMFCS) that classifies the child’s functional abilities in five levels (7). However, the trend is the increased use of objective instruments such as: force platforms; electrogoniometry wi-fi, electromyography, mechanomyography, and dual-energy x-ray absorptiometry (DEXA), in monitoring of nutritional status and fat free mass (3).

## Conclusion

The intensive physical therapy protocols with special clothes associated with objective instruments of evaluation and functional diagnosis has proven to be a trend in global neurology rehabilitation. These protocols can combine the best elements of various techniques and therapeutic methods and, is well supported by the studies physiology of the exercise. Although one cannot clarify the influence of the suit on the success of the treatment protocol, the suit makes it easy the conduction of the treatment by the therapist. In a short time, conventional physiotherapy (few hours of treatment per week) is no longer a common practice for neuropediatric patients. Technological advances and the use of objective assessment tools allows more precision in planning treatment protocol.
